# Estimation of Abbreviated Cyclosporine A Area under the Concentration-Time Curve in Allogenic Stem Cell Transplantation after Oral Administration

**DOI:** 10.1155/2012/342701

**Published:** 2011-10-26

**Authors:** Hanene ELjebari, Nadia Ben Fradj, Issam Salouage, Emna Gaies, Sameh Trabelsi, Nadia Jebabli, Mohamed Lakhal, Tarek Ben Othman, Anis Kouz

**Affiliations:** ^1^Laboratoire de Pharmacologie Clinique, Centre National de Pharmacovigilance, 9 Avenue Dr. Zouheïr Essafi, Tunis 1006, Tunisia; ^2^Faculté de Médecine de Tunis, 15 Rue Djebel Lakhdar La Rabta, Tunis 1007, Tunisia; ^3^Centre National de Greffe de Moelle Osseuse, Rue Djebel Lakhdar La Rabta, Tunis 1007, Tunisia

## Abstract

Measurements of Cyclosporine (CsA) systemic exposure permit its dose adjustment in allogenic stem cell transplantation recipients to prevent graft-versus-host disease. CsA LSSs were developed and validated from 60 ASCT patients via multiple linear regressions. All whole-blood samples were analyzed by fluorescence polarization immunoassay (FPIA-Axym). The 10 models that have used CsA concentrations at a single time point did not have a good fit with AUC_0–12_ (R^2^ < 0.90). *C*_2_ and *C*_4_ were the time points that correlated best with AUC_0–12 h_, R^2^ were respectively 0.848, and 0.897. The LSS equation with the best predictive performance (bias, precision and number of samples) utilized three sampling concentrations was AUC_0–12 h_ = 0.607 + 1.569 × *C*_0.5_ + 2.098 × *C*_2_ + 3.603 × *C*_4_ (R^2^ = 0.943). Optimal LSSs equations which limited to those utilizing three timed concentrations taken within 4 hours post-dose developed from ASCT recipient's patients yielded a low bias <5% ranged from 1.27% to 2.68% and good precision <15% ranged from 9.60% and 11.02%. We propose an LSS model with equation AUC_0–12 h_ = 0.82 + 2.766 × *C*_2_ + 3.409 × *C*_4_ for a practical reason. Bias and precision for this model are respectively 2.68% and 11.02%.

## 1. Introduction 

Cyclosporine is one of the most common immunosuppressants used in allogenic stem cells transplant (ASCT) both in children and adults [1, 2]. This population had more digestive problems compared to solid transplant patient; consisting of mucositis and diffuse inflammation of the intestinal tract related to the preparative regimen. In addition, they frequently develop digestive graft-versus-host disease (GVHD) and intestinal viral disease affecting the absorption of CsA [3–5]. In most case cyclosporine is administered intravenously from day −1 to days 30–50 then orally until day 180 [6].

Individualization of cyclosporine (CsA) dosage using therapeutic drug monitoring remains an indispensable tool for management of CsA therapy [7, 8]. A number of clinical trials have employed *C*_2_ monitoring in solid organ transplantations and demonstrated improvements in rejection rates and acute toxicity compared to trough-level (*C*_0_) monitoring [9, 10].

In ASCT Barkholt et al. [11] have not found a correlation between *C*_2_ and either severs GVHD or infection in ASCT patients with *C*_0_ guided CsA dosing. Furukawa et al., 2010 [12], demonstrate that in ASCT patients receiving twice-daily 3 h intravenous administrations, the concentration of CsA at 2 h (*C*_2_) and 2 h and 50 min (*C*_3_) were best single predictors of exposure (AUC_0–12 h_) while the trough concentration correlates poorly. Inoue et al. [13] were observed two close relationships between AUC_0–12 h_ and the *C*_3_ for infusion and between AUC_0–12 h_ and the *C*_8_ for oral administration.

The area under a blood drug concentration-time curve (AUC) is an important index in therapeutic drug monitoring and in pharmacokinetics. Unfortunately, obtaining complete pharmacokinetic profiles from transplant patients is not always an easy task due to ethical, practical, and economical reasons. Therefore, reducing the number of blood samples necessary for determinations would be very practical for patient management and would help in cyclosporine monitoring. Recently, limited sampling strategies (LSSs) have been developed to predict the AUC_0–12 h_; several of them are used to predict cyclosporine AUC in transplanted patients. Up to now, more than 100 equations to estimate AUC using limited sampling strategy have been published, but most of them have not been validated [14]. 

ASCT outcomes may be optimized by cyclosporine dose adjustment according to systemic exposure (measured by AUC) rather than trough concentration. 

The aim of this study was to develop LSSs for predicting cyclosporine AUC_0–12 h_ to provide a practical method for a more accurate therapeutic (TDM) after oral dosing in patients undergoing ASCT and validated selected models.

## 2. Patients and Methods 

### 2.1. Patients

Following ethics approval by the institutional review board, 60 ASCT recipient's patients enrolled the study (new graft in 2009). The patient's characteristics are listed in Table 1. Diseases consisted in acute myeloblastic leukemia (*n* = 32 patients) acquired aplastic anemia (*n* = 12 patients), acute lymphoblastic leukemia (*n* = 8), Fanconi's anemia (*n* = 4 patients), multiple myeloma (*n* = 1 patient), and Gaucher disease (*n* = 1 patient). 

Conditioning regimens consisted on IV Busulfan + Cyclophosphamide (36 patients), Fractionated Total body Irradiation + Etoposide (8 patients), horse antithymocyte globulin + Cyclophosphamide (12 patients), Fludarabine + Cyclophosphamide (4 patients). All patients received oral antimicrobial prophylaxis with amoxicillin or spiramycin, fluconazole, and acyclovir. 

CsA (Sandimmun) was started by continuous intravenous (i.v.) infusion from day 1 to 15–21 days after transplantation, and then patients were randomized to oral CsA (equoral or neoral) at twice IV dose. After that all patients received oral CsA (mean of 300 mg/day). The daily dose was taken in two equally divided doses at 12 h intervals. 

### 2.2. Inclusion and Exclusion Criteria 

After the conditioning regimes, patients were included in the study, if they were aged between 3 and 50 years, clinically stable after a first ASCT, and receiving oral CsA as graft-versus-host disease prophylaxis. Patients should have stable serum creatinine (<2 baseline value) and no history of hepatic dysfunction (bilirubin and aminotransferases <2 normal value). 

Patients excluded if they developed graft-versus-host disease or microangiopathy, received another drug which interferes with CsA pharmacokinetics, and showed evidence of noncontrol digestive problem, renal (2 × creatinine baseline value), or hepatic dysfunction (bilirubin and aminotransferases >2 × normal value).

### 2.3. Blood Sampling and Measurements of Cyclosporine Concentrations

After written informed consent, blood samples (3 mL) were collected in tubes containing EDTA anticoagulant from the patients at time 0 (*C*_0_; pre dose) and at 0.5 h (*C*_0.5_), 1 h (*C*_1_), 1.5 h (*C*_1.5_), 2 h (*C*_2_), 3 h (*C*_3_), 4 h (*C*_4_), 6 h (*C*_6_), 8 h (*C*_8_), and 12 h (*C*_12_) after steady-state morning Cs dose. All whole-blood samples were analyzed by fluorescence polarization immunoassay (FPIA-Axym) in Laboratory of Clinical Pharmacology of the National Center of Pharmacovigilance. Precision data of immunoassay was demonstrated previously by comparison of the two methods for CsA therapeutic monitoring HPLC and AxSYM. Result showed that the correlation coefficient is 0.99 [15].

### 2.4. Pharmacokinetic and Statistical Analysis

#### 2.4.1. Pharmacokinetics

Peak CsA concentration (C_max_) and the time to reach it (T_max_) were recorded for each patient and were reported as the mean ± standard deviation. 

Pharmacokinetic parameters were calculated using a noncompartment model. The area under the plasma concentration-time curves AUC_0–12 h_ was calculated from drug plasma concentration data based on trapezoid rule [16].

#### 2.4.2. Limited Sampling Strategy (LSS)

CsA LSSs were developed and validated from 60 ASCT patients (30 in the development group and 30 in the validation group) via multiple linear regressions.

**Step 1**

Multiple linear regressions were performed to estimate abbreviated AUC (dependent variable) and each time point of CsA levels (independent variables) that best fitted the CsA AUC_0–12 h_. Selected models are those who have *P* < 0.05 for any sampling time and high correlation coefficient (*R* ^2^ > 0.9). These analyses produced equations AUC as follows: AUC = Cst + *M*_1_ × *C*_1_ + *M*_2_ × *C*_2_ + ⋯+*M*_*n*_ × *C*_*n*_; *M* and Cst are coefficients and *n* is the number of samples.

Optimal LSS equations were limited to those utilizing a maximum of four timed concentrations taken within 6 hours after dose, and with a coefficient of determination (*R* ^2^) > 0.9.

**Step 2**

To validate the equation, we used a second group of pharmacokinetic profile data obtained from ASCT patients recipients (validation group) with the same characteristics as the initial group. Predictive performances of LSS equations were evaluated by calculating the percentage bias (mean prediction error me %) and percentage precision (root-mean-squared prediction error rmse %) [17, 18].

(1) $$\begin{array}{c} \text{me\%}=\frac{\sum_{}^{}\left(\text{\%}\;\text{pei}\right)}{N},\\ \text{rmse\%}=\sqrt{\left(\frac{\Sigma \;{\left(\text{\%}\;\text{pei}\right)}^{2}}{N}\right)}, \end{array}$$

where *N* is the number of AUC pairs and (% pei) on the AUC, defined as ([AUC_predicted_ − AUC_observed_]/AUC_observed_)∗100 and (% rmse). A positive or negative me_*i*_ indicates, respectively, overprediction or underprediction of the concentrations by the model. Precision and mean bias < 15% was acceptable [8].

### 2.5. Statistical Analysis

The association between cyclosporine AUC using all available data points (AUC-all) and the AUC values predicted by each LSS was described using Pearson correlation coefficients (*R* ^2^); values >0.9 were acceptable. Multiple stepwise regression analysis was used to determine the points of CsA samples that derived model equations that best fitted the CsA AUC_0–12 h_. 

All statistical analyses were performed using SPSS software for windows (version 11.5.0 Inc., Chicago, Illinois). Data were expressed as mean ± SD.

## 3. Results

**Figure 1:**
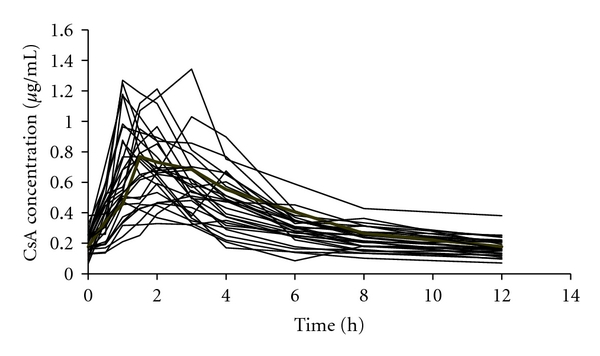
Individual blood concentration-time curves of patients administered CsA (*n* = 30).

Thirty full pharmacokinetic profiles of CsA were obtained (Figure 1). The mean CsA dose at the time of the measurement was 113 ± 40.85 mg. The peak concentration of CsA generally accrued 2-3 hours after morning dosing. Mean trough concentration *C*_0_ was 0.470 ± 0.178 *μ*g·mL^−1^. The mean AUC_0–12 h_ was 4.340 ± 1.195 *μ*g·h·mL^−1^ (Table 2).

**Figure 2:**
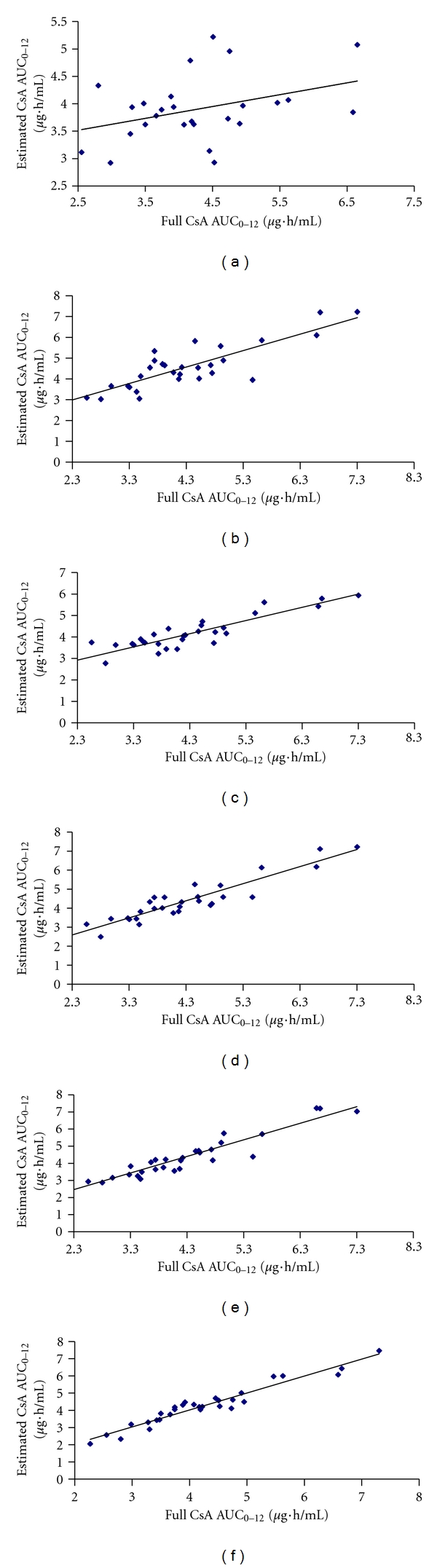
Comparison of the full cyclosporine (CsA) AUC_0–12 h_ and values estimated using 6 regression models: (a) model using trough concentration (*R* ^²^ = 0.391), (b) model using concentration at 2 hours after dosing (*R* ^2^ = 0.848), (c) model using concentration at 4 hours after dosing (*R* ^2^ = 0.897), (d) model using concentrations at 2 and 4 hours after dosing (*R* ^2^ = 0.930), (e) model using concentrations at 0.5, 2 and 4 hours after dosing (*R* ^2^ = 0.943), and (f) model using concentrations at 2, 4, 6 and 8 hours after dosing (*R* ^2^ = 0.965).

The linear regression equations and the concentration at each time point are listed in Table 3. The 10 models that used CsA concentrations at a single time point did not have a good fit (*R* ^2^ < 0.90). *C*_2_ and *C*_4_ were the time points that correlated best with AUC_0–12 h_, *R* ^2^ were, respectively, 0.848 and 0.897. Moreover, the correlation between AUC_0–12 h_ and the trough concentration (*C*_0_ or *C*_12_) was poor, *R* ^2^ = 0.391 for *C*_0_ and *R* ^2^ = 0.786 for *C*_12_ (Table 3). The correlations between AUC_0–12 h_ and CsA concentrations at various points *C*_0_, *C*_2_, and *C*_4_, are demonstrated in Figures 2(a), 2(b), and 2(c). The dose of CsA per kilogram of body weight and total dose of CsA did not correlate with AUC_0–12 h_ (data not shown).

Pharmacokinetic data from 30 ASCT recipient's patients were used to test the LSS equations. Multiple linear regression analysis of the correlation between the estimated cyclosporine (CsA) AUC_0–12 h_ and full hours CsA AUC were listed in Table 4. 

**Figure 3:**
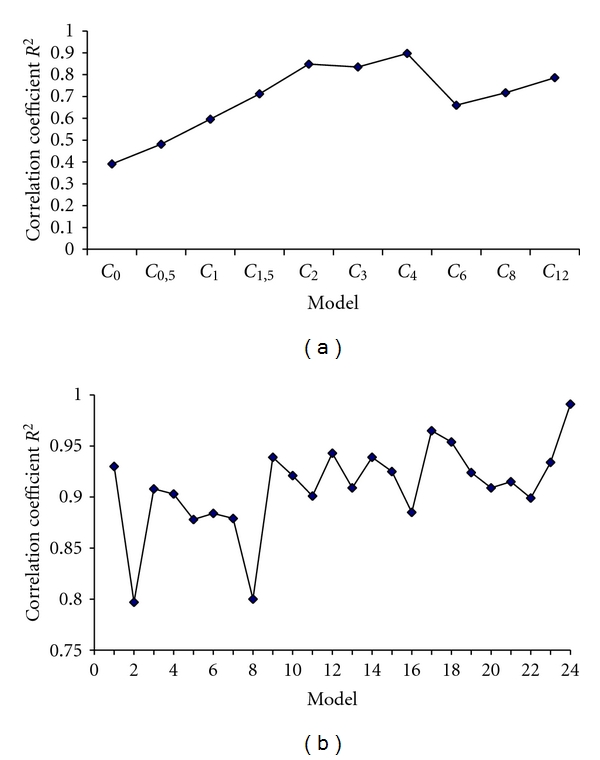
Evolution of Correlation coefficients *R* ^2^ according selected models. (a) Correlation coefficients between AUC_0–12 h_ and CsA concentration at each time point. (b) Correlation coefficients between AUC_0–12 h_ estimated and concentrations at full hours.

From the various equations obtained for the estimation of CsA AUC, the equation delineated from all of the concentration-time data (AUC_0–12 h_ = 0.152 − 2.249 × *C*_0_+0.577 × *C*_0.5_+0.871 × *C*_1_−0.001 × *C*_1.5_+  0.963 × *C*_2_+1.16 × *C*_3_+0.152 × *C*_4_+1.66 × *C*_6_+3.47 × *C*_8_+2.703 × *C*_12_; *R* ^2^ = 0.991) had the highest coefficient of determination (Figure 3(a)). 

Among models that used CsA concentrations at 2 time points, best model **1 **(*R* ^2^ = 0.930), which used *C*_2_ and *C*_4_. Among models that used CsA concentrations at 3 time points, best model **12 **(*R* ^2^ = 0.943), which used *C*_0.5_, *C*_2_, and *C*_4_. Among models that used CsA concentrations at 4 time points, best model **17 **(*R* ^2^ = 0.965), which used *C*_2_, *C*_4_, *C*_6_, and *C*_8_ (Figure 3(b)). However, the negative bias obtained for this combination (−0.21%) indicates that it tends to underpredict AUC (Figure 2(f)). Optimal LSS equations were limited to those utilizing a maximum of three timed concentrations taken within 4 hours after dose, and with a coefficient of determination (*R* ^2^ > 0.9):

(2) $$\begin{array}{cc} {\text{AUC}}_{0\text{–}12\;\text{h}} & =0.82+2.766\times C_{2}+3.409\times C_{4}\;\left(R^{2}=0.930\right), \end{array}$$

(3) $$\begin{array}{cc} {\text{AUC}}_{0\text{–}12\;\text{h}} & =0.735+0.388\times C_{1}+2.357\times C_{2}\\ & \;+3.654\times C_{4}\;\left(R^{2}=0.939\right), \end{array}$$

(4) $$\begin{array}{cc} {\text{AUC}}_{0\text{-}12\;\text{h}} & =0.607+1.569\times C_{0.5}+2.098\times C_{2}\\ & \;+3.603\times C_{4}\;\left(R^{2}=\;0.943\right). \end{array}$$

Predictive performance of all LSSs models were summarized in Table 5. All three optimal LSSs equations developed from ASCT recipient's patients in our laboratory yielded a low bias <5% ranged from 1.27% to 2.68% and good precision <15% ranged from 9.60% and 11.02%. The LSS equation with the best predictive performance (bias, precision, and number of samples) was (4), which utilizes *C*_0.5_, *C*_2_, and *C*_4_ (Figure 2(e)). The LSS equation (2) utilizes *C*_2_ and *C*_4_ demonstrated low bias (2.68%) and good precision (11.02%; Figure 2(d)).

## 4. Discussion 

Graft-versus-host disease (GVHD) remains a major limiting factor for a successful result after ASCT; it affects mortality, morbidity, and quality of life. CsA-based immunosuppression has been the most frequently used regimen for prophylaxis and treatment of GVHD in patients undergoing HSCT [19, 20]. Therapeutic monitoring of CsA provides a more accurate measure of posttransplant immunosuppression and this by assessment of pharmacokinetic parameters. The area under concentration-time curve (AUC) is a more important index in therapeutic drug monitoring and in pharmacokinetics. In this investigation patients were received a mean dose of cyclosporine A (neoral or equoral) 113.0 ± 40.85 mg. A recent study demonstrates that two formulations were bioequivalent in ASCT patients [21]. 

The time to reach C_max _(T_max_) in this study was 1.908 ± 0.954; this is similar to the values reported for other HSCT patients, 1.9 ± 0.8 h [22] 2.4 ± 1.1 h [3]. As previous studies have reported, T_max _ for HSCT recipients has shown that absorption is delayed in comparison to that measured in solid organ allograft recipients [22]. It is thought that the differences are due to the presence of gastrointestinal inflammation caused by mucositis or GVHD [3].

A number of trials have employed *C*_2_ monitoring in solid organ transplantation [23], *C*_2_ monitoring is being increasingly employed in the management of solid organ recipients with a recent survey of renal transplant centres finding that most of the respondents were now measuring *C*_2_ concentration [24]. In a stem cells transplant setting, where it is clear that current practice of trough-level monitoring still results in unacceptably high levels of GVHD, therefore is a scope to explore the potential of monitoring the drug (both I.V and oral) via alternative measures of exposure or activity [1]. A recent investigation demonstrate a close relationship between AUC_0–12 h_ and the *C*_8_ after oral administration of CsA in allogenic haematopoietic stem cell transplantation (ASTH) [13], and between AUC_0–12 h_ and two concentrations *C*_3_ and *C*_2_ after twice-daily infusion [12, 13]. 

Therapeutic monitoring of Tunisian ASTH patients was based on *C*_0_ determination; this concentration is weakly correlated with AUC_0–12 h_  (*R* ^2^ = 0.391). That we bring to search what concentrations are well correlated with AUC and how to estimate abbreviated AUC in ASCT.

The major findings of this study are that the concentration measurements at *C*_2_ and *C*_4_ were the time points that correlated best with AUC_0–12 h_ after oral administration of CsA in allogenic stem cells transplant patients rather than the trough level. These time points provide a more accurate measure of posttransplant immunosuppression. Therefore, the target concentration of CsA in ASHT patients, after the switch to oral administration using *C*_2_ monitoring to adjust the dose, is easily applied the same level of *C*_4_ monitoring with oral administration. 

Another clinically attractive finding of our study was using two time sampling points *C*_2_ and *C*_4_ for estimating CsA AUC_0–12 h_. The regression equation was AUC_0–12 h_ = 0.82 + 2.766 × *C*_2_ + 3.409 × *C*_4_, *R* ^2^ = 0.930. The bias of this model was 2.68%, and the precision was 11.02%. Additional *C*_4_ sampling is valuable to support the individual dosing. To further increase the accuracy and decrease bias of predicted AUC_0–12 h_, we added time point sampling in absorption phase *C*_0.5_ (*R* ^2^ = 0.943, me% = 1.27, rmse% = 9.60) or *C*_1_ (*R* ^2^ = 0.939, me% = 1.35, rmse% = 9.76). Indeed, optimal LSSs equations, which are limited to those utilizing three timed concentrations taken within 4 hours after dose, are (4) and (3). Optimal Among models that used CsA concentrations at 4 time points are model 17 (AUC_0–12 h_ = 0.148 + 2.457 × *C*_2_ + 1.582 × *C*_4_ + 1.296 × *C*_6_ + 5.81 × *C*_8_, *R* ^2^ = 0.965). However, the negative bias obtained for this combination (−0.21%) indicates that it tends to underpredict AUC.

## 5. Conclusion 

Limited sampling strategy models were selected to predict the full 12-hour CsA AUC in allogenic haematopoietic stem cell transplant patients receiving CsA. We propose an LSS model with equation AUC_0–12 h_ = 0.82 + 2.766 × *C*_2_ + 3.409 × *C*_4_, which is limited to utilizing two timed concentrations taken within 4 hours after dose and providing a low bias and a good precision, it is for a practical reason.

## Figures and Tables

**Table 1:** Characteristics and laboratory test results of allogenic hematopoietic stem cell transplantation (*N* = 60).

Patients characteristics	Mean ± SD
Age (yrs)	28.09 ± 11.79
Weight (Kg)	55.5 ± 16.40

Laboratory test results	
Serum creatinine (mmol/L)	48.73 ± 17.78
Biirubin (mg/L)	12.0 ± 4.39
Alkaline phosphatase (UI/L)	122.31 ± 80.43
AST (UI/L)	45.21 ± 83.37
ALT (UI/L)	52.55 ± 111.20

SD: standard deviation.

**Table 2:** Pharmacokinetic parameters (PK) in allogenic hematopoitic stem cell transplantation.

PK parameters (LSS development group)	Values (Mean ± SD)
AUC_0–12 h_ (*μ*g·h·mL^−1^)	4.340 ± 1.195
Through concentration C_0 _(*μ*g·mL^−1^)	0.470 ± 0.178
C_max_ (*μ*g·mL^−1^)	0.834 ± 0.279
T_max_ (h)	1.908 ± 0.954
Dose (mg)	113.0 ± 40.85

AUC_0–12 h_, area under the concentration-time curve from 0–12 h; C_max_, peak concentration, T_max_, time to reach C_max_; SD, standard deviation.

**Table 3:** Correlations and regression equations between AUC_0–12 h_ and CsA concentration at each time point.

Sampling time (h)	Model equation for abbreviated AUC (*μ*g·h/mL)	Pearson *R* ^2^
0	AUC_0–12 h_ = 1.59 + 13.371 × *C*_0_	0.391
0.5	AUC_0–12 h_ = 2.779 + 3.197 × *C*_0.5_	0.481
1	AUC_0–12 h_ = 3.332 + 1.558 × *C*_1_	0.596
1.5	AUC_0–12 h_ = 1.797 + 3.639 × *C*_1.5_	0.712
2	AUC_0–12 h_ = 1.384 + 4.268 × *C*_2_	0.848
3	AUC_0–12 h_ = 1.52 + 4.76 × *C*_3_	0.835
4	AUC_0–12 h_ = 1.818 + 5.357 × *C*_4_	0.897
6	AUC_0–12 h_ = 1.479 + 9.651 × *C*_6_	0.660
8	AUC_0–12 h_ = 1.37 + 12.683 × *C*_8_	0.717
12	AUC_0–12 h_ = 1.772 + 14.489 × *C*_12_	0.786

*C*_*x*_ concentration at *x* time, AUC area under the concentration-time curve.

**Table 4:** Multiple linear regression analysis of the correlation between the estimated cyclosporine (CsA) AUC_0–12 h_ and full hours CsA AUC (*N* = 30).

Models	Sampling time (h)	Model equation for abbreviated AUC	Pearson *R* ^2^
**1**	2, 4	AUC_0–12 h_ = 0.82 + 2.766 × *C*_2_ + 3.409 × *C*_4_	0.930
**2**	0, 2	AUC_0–12 h_ = 0.697 + 7.85 × *C*_0_ + 2.926 × *C*_2_	0.797
**3**	2, 6	AUC_0–12 h_ = 0.483 + 2.752 × *C*_2_ + 6.58 × *C*_6_	0.908
**4**	2, 3	AUC_0–12 h_ = 0.936 + 2.012 × *C*_2_ + 3.405 × *C*_3_	0.903
**5**	3, 4	AUC_0–12 h_ = 1.364 + 3.694 × *C*_3_ + 1.688 × *C*_4_	0.878
**6**	3, 6	AUC_0–12 h_ = 1.305 + 3.534 × *C*_3_ + 3.199 × *C*_6_	0.884
**7**	0, 2, 6	AUC_0–12 h_ = 0.291 + 3.972 × *C*_0_ + 2.336 × *C*_2_ + 5.44 × *C*_6_	0.879
**8**	0, 4, 6	AUC_0–12 h_ = 0.768 + 6.412 × *C*_0_ + 2.172 × *C*_4_ + 4.152 × *C*_6_	0.80
**9**	1, 2, 4	AUC_0–12 h_ = 0.735 + 0.388 × *C*_1_ + 2.357 × *C*_2_ + 3.654 × *C*_4_	0.939
**10**	1.5, 3, 4	AUC_0–12 h_ = 0.701 + 1.924 × *C*_1.5_ + 2.545 × *C*_3_ + 1.682 × *C*_4_	0.921
**11**	1.5, 2, 4	AUC_0–12 h_ = 0.733 + 2.833 × *C*_1.5_ + 0.252 × *C*_2_ + 3.818 × *C*_4_	0.901
**12**	0.5, 2, 4	AUC_0–12 h_ = 0.607 + 1.569 × *C*_0.5_ + 2.098 × *C*_2_ + 3.603 × *C*_4_	0.943
**13**	0, 2, 4	AUC_0–12 h_ = 0.464 + 5.388 × *C*_0_ + 2.155 × *C*_2_ + 2.708 × *C*_4_	0.909
**14**	2, 4, 6	AUC_0–12 h_ = 0.471 + 2.51 × *C*_2_ + 1.367 × *C*_4_ + 5.015 × *C*_6_	0.939
**15**	2, 3, 4	AUC_0–12 h_ = 0.807 + 1.952 × *C*_2_ + 2.495 × *C*_3_ + 1.504 × *C*_4_	0.925
**16**	1, 1.5, 2, 4	AUC_0–12 h_ = 0.842 − 0.372 × *C*_1_ + 2.834 × *C*_1.5_ + 0.036 × *C*_2_ + 3.681 × *C*_4_	0.885
**17**	2, 4, 6, 8	AUC_0–12 h_ = 0.148 + 2.457 × *C*_2_ + 1.582 × *C*_4_ + 1.296 × *C*_6_ + 5.81 × *C*_8_	0.965
**18**	1, 2, 4, 6	AUC_0–12 h_ = 0.23 + 0.851 × *C*_1_ + 1.573 × *C*_2_ + 1.57 × *C*_4_ + 5.838 × *C*_6_	0.954
**19**	0, 2, 4, 6	AUC_0–12 h_ = 0.287 + 3.821 × *C*_0_ + 2.127 × *C*_2_ + 1.271 × *C*_4_ + 4.033 × *C*_6_	0.924
**20**	1.5, 2, 3, 4	AUC_0–12 h_ = 0.75 + 2.726 × *C*_1.5_ − 1.138 × *C*_2_ + 2.764 × *C*_3_ + 1.787 × *C*_4_	0.909
**21**	0.5, 1.5, 2, 4	AUC_0–12 h_ = 0.731 + 0.365 × *C*_0.5_ + 1.895 × *C*_1.5_ + 0.524 × *C*_2_ + 3.793 × *C*_4_	0.915
**22**	0, 1.5, 2, 4	AUC_0–12 h_ = 0.483 + 4.604 × *C*_0_ + 1.613 × *C*_1.5_ + 0.467 × *C*_2_ + 3.099 × *C*_4_	0.899
**23**	1.5, 2, 4, 6	AUC_0–12 h_ = 0.414 + 2.401 × *C*_1.5_ − 0.141 × *C*_2_ − 1.748 × *C*_4_ + 5.137 × *C*_6_	0.934
**24**	Full hours	AUC_0–12 h_ = 0.152 − 2.249 × *C*_0_ + 0.577 × *C*_0.5_ + 0.871 × *C*_1_ − 0.001 × *C*_1.5_ + 0.963 × *C*_2_ + 1.16 × *C*_3_ + 0.152 × *C*_4_ + 1.66 × *C*_6_ + 3.47 × *C*_8_ + 2.703 × *C*_12_	0.991

**Table 5:** Prediction bias and precision of models for estimating the cyclosporine (CsA) AUC_0–12 h_ (*n* = 30).

Models	Sampling time (h)	Bias (me%)	Precision (rmse%)
**1**	2, 4	2.68	11.02
**2**	0, 2	−0.09	11.74
**3**	2, 6	5.48	13.63
**4**	2, 3	4.10	13.08
**5**	3, 4	0.97	13.97
**6**	3, 6	2.64	13.28
**7**	0, 2, 6	−0.60	10.64
**8**	0, 4, 6	−4.95	15.76
**9**	1, 2, 4	1.35	9.76
**10**	1.5, 3, 4	1.30	11.65
**11**	1.5, 2, 4	0.69	13.57
**12**	0.5, 2, 4	1.27	9.60
**13**	0, 2, 4	−2.42	9.54
**14**	2, 4, 6	2.83	9.61
**15**	2, 3, 4	2.22	11.33
**16**	1, 1.5, 2, 4	0.58	15.05
**17**	2, 4, 6, 8	−0.21	8.04
**18**	1, 2, 4, 6	2.59	8.50
**19**	0, 2, 4, 6	−0.60	8.77
**20**	1.5, 2, 3, 4	41.77	45.19
**21**	0.5, 1.5, 2, 4	2.39	12.55
**22**	0, 1.5, 2, 4	−1.25	10.07
**23**	1.5, 2, 4, 6	3.6	10.78
**24**	All	2.41	2.86
Mean IC 95%		2.86 (−0.57–6.29)	12.49 (9.51–15.48)
